# A lowered 26S proteasome activity correlates with mantle lymphoma cell lines resistance to genotoxic stress

**DOI:** 10.1186/s12885-017-3530-z

**Published:** 2017-08-10

**Authors:** Khaoula Ben Younes, Simon Body, Élodie Costé, Pierre-Julien Viailly, Hadjer Miloudi, Clémence Coudre, Fabrice Jardin, Fatma Ben Aissa-Fennira, Brigitte Sola

**Affiliations:** 1 Normandie Univ, INSERM UMR 1245, UNIROUEN, UNICAEN, Caen, France; 2 Faculté de médecine, Laboratoire de Génétique, d’Immunologie et de Pathologie humaines, Université de Tunis El Manar, Tunis, Tunisia; 3 Département d’Hématologie Clinique, Centre de Lutte contre le Cancer Henri Becquerel, Rouen, France; 4 MICAH, UFR Santé, CHU Côte de Nacre, 14032 Caen Cedex, France

**Keywords:** B-cell lymphoma, Apoptosis, Cell cycle, Senescence, Resistance/sensitivity, Double-strand break, DNA repair, Ubiquitin ligase, 26S proteasome, PSMB6

## Abstract

**Background:**

Mantle cell lymphoma (MCL) is a B-cell hemopathy characterized by the t(11;14) translocation and the aberrant overexpression of cyclin D1. This results in an unrestrained cell proliferation. Other genetic alterations are common in MCL cells such as SOX11 expression, mutations of *ATM* and/or *TP53* genes, activation of the NF-κB signaling pathway and NOTCH receptors. These alterations lead to the deregulation of the apoptotic machinery and resistance to drugs. We observed that among a panel of MCL cell lines, REC1 cells were resistant towards genotoxic stress. We studied the molecular basis of this resistance.

**Methods:**

We analyzed the cell response regarding apoptosis, senescence, cell cycle arrest, DNA damage response and finally the 26S proteasome activity following a genotoxic treatment that causes double strand DNA breaks.

**Results:**

MCL cell lines displayed various sensitivity/resistance towards genotoxic stress and, in particular, REC1 cells did not enter apoptosis or senescence after an etoposide treatment. Moreover, the G2/M cell cycle checkpoint was deficient in REC1 cells. We observed that three main actors of apoptosis, senescence and cell cycle regulation (cyclin D1, MCL1 and CDC25A) failed to be degraded by the proteasome machinery in REC1 cells. We ruled out a default of the βTrCP E3-ubiquitine ligase but detected a lowered 26S proteasome activity in REC1 cells compared to other cell lines.

**Conclusion:**

The resistance of MCL cells to genotoxic stress correlates with a low 26S proteasome activity. This could represent a relevant biomarker for a subtype of MCL patients with a poor response to therapies and a high risk of relapse.

**Electronic supplementary material:**

The online version of this article (doi:10.1186/s12885-017-3530-z) contains supplementary material, which is available to authorized users.

## Background

Mantle cell lymphoma (MCL) is an aggressive lymphoid malignancy derived from mature B cells, characterized by a rapid clinical evolution and a poor response to current therapies [1]. The first oncogenic hit for tumor development is the translocation t(11;14)(q13;q32) which juxtaposes activating sequences from the *IGH* gene promoter upstream of the *CCND1* gene. This translocation leads to the constant expression of cyclin D1 protein and in turn, abnormalities of cell cycle, and compromises the G1-S checkpoint [1]. This initial oncogenic event is followed by various chromosomal alterations targeting DNA damage response (DDR), survival pathways, NOTCH and NF-κB pathways, and chromatin modification machinery [2] as well as reprograming metabolism [3].

ATM (Ataxia telangectasia mutant) and ATR (ATM and Rad3-related) act as apical kinases and key regulators of DDR. Following double-strand DNA breaks (DSBs), ATM/ATR phosphorylate downstream effectors including checkpoint kinases (CHK1/CHK2), DNA repairing factors and transcriptional regulators such as p53 [4]. Next, depending on the cellular context, cells initiate cell cycle arrest, DNA repair through two main mechanisms: homologous recombination (HR) or non-homologous end joining (NHEJ), and/or apoptosis. *ATM* alterations are very common in MCL patients, mutations and deletions occurring in up to half of cases [5]. Genetic alterations of *TP53* are also very common (30% of cases) and concurrent alterations of *ATM* and *TP53* are found in almost 10% of patients [6]. Defaults in responding intracellular and extracellular genotoxic stresses could explain why MCL is the B-cell malignancy with the highest degree of genomic instability [7].

Abnormalities of the ubiquitin-proteasome pathway are also recognized in MCL cells. They could account for defaults in the DDR and resistance towards genotoxic drugs that are used in clinics such as cyclophosphamide, doxorubicin and chlorambucil [8]. For example, MCL cells show frequent deletion within the *FBXO25* gene located at 8p23.3 [9]. *FBXO25* encodes a F-box containing protein, part of the Skp1/Cullin/F-box containing protein or SCF^FBXO25^ complex that targets the prosurvival HAX1 mitochondrial protein. The monoallelic loss of *FBXO25* and thus, the disruption of the PRKCD (a protein kinase C)/FBXO25/HAX1 axis promotes survival of MCL cells. A high percentage of MCL tumors (20%) have mutations within the *UBR5* gene [10]. UBR5 encodes an E3 ubiquitin ligase that targets KATNA1 (katanin p60), TOPBP1 (DNA topoisomease 2-binding protein 1) and PAIP2 (polyadenylate-binding protein-interacting protein 2) proteins whose functions are not fully known. The human double minute(HDM)-2 E3 ubiquitin ligase plays a key role in p53 turnover. The gene is located within the 12q13 locus which is amplified in MCL [11]. This accounts for elevated HDM2 expression and prevention of both p53 transcriptional activity and degradation. Thus, the response of MCL cells to DNA damaging agents is impaired through various mechanisms.

Studying a set of MCL cell lines, we noticed that REC1 cells were particularly resistant to genotoxic stresses. Looking for cellular mechanisms that could sustain this resistance, we observed that the ubiquitin/proteasome degradation pathway was inefficient. We ruled out a default of β-transducin repeat containing protein (βTrCP), the E3 ubiquitin ligase of the SCF^βTrCP^ complex which was a good candidate. We further used fluorescent probes to study specifically the 26S proteasome activity and observed that this activity was specifically down-regulated in REC1 cells compared to other MCL cell lines.

## Methods

### Cell cultures, treatments and cell proliferation determination

MCL cell lines were provided by Gaël Roué (IDIBAPS, Barcelona, Spain) except Granta519 cells which were purchased from DSMZ (ACC-342). MCL cell lines were maintained in culture as described [12]. Cell authentication was done by STR profiling (IdentiCell, Aarhus, Denmark). Cell proliferation was analyzed using the CellTiter 96® AQueous One Solution Cell Proliferation assay (Promega, Charbonnières, France) according to the supplier. MCL cells were treated with vehicle (0.01% DMSO) or 1–40 μg/ml etoposide (Sigma-Aldrich, St Louis, MO) for 24–72 h depending on the experiment. For co-treatment with MG132, the cells were incubated with 5 μM MG132 (Sigma-Aldrich) together with 4 μg/ml etoposide for 24 h.

### Quantification of apoptotic and senescent cells, cell cycle analysis

MCL cells exposed to vehicle or etoposide were stained with an Apo 2.7 PE-conjugated antibody (Ab, Beckman Coulter, Villepinte, France). The APO2.7-stained cells were analyzed by flow cytometry (Gallios, Beckman Coulter) and data were processed with the Kaluza software (Beckman Coulter). At least, 2 × 10^4^ cells were analyzed for each culture condition, for each experiment.

For cell cycle experiments, the cells were washed with PBS and fixed in 70% ethanol/PBS at −20 °C for 30 min. After washing, the cells were then incubated with PBS containing 10 mg/ml of propidium iodide (PI) and 100 mg/ml of RNAse A. At least, 2 × 10^4^ cells were analyzed by flow cytometry for each experimental condition.

To assess the presence of senescent cells after etoposide treatment (4 μg/ml for 24 h), we used a cytometry-based assay after staining of living cells with 5-dodecanoylaminofluorescein di-β-D-galactopyranoside (C_12_FDG) as described previously [13]. A shift of the mean fluorescence intensity (MFI) is representative of an enrichment of senescent cells in the whole population.

### Indirect immunofluorescence and confocal fluorescence microscopy analysis

Cells (10^5^ cells per spot) were cytospun on Superfrost glass slides, at 500 x g for 3 min, then fixed in 4% paraformaldehyde (PFA) for 15 min, and permeabilized by incubation with 0.5% Triton-X100 (*v*/v) for 5 min. The slides were then incubated with 3% BSA in PBS for 30 min at room temperature and, next with Abs anti-γH2AX (p-S139, final dilution 1/2000) (GTX61796 from GeneTex Inc., Irvine, CA) or anti-cyclin D1 (sc-718, Santa Cruz Tech., final dilution 1/50) for overnight in the dark at 4 °C. Goat anti-rabbit IgG (H + L) polyclonal Alexa Fluor® 546 (Life Technologies) served as secondary Ab. Following staining steps, cells were mounted with VECTASHIELD® with DAPI (Vector Lab.). The slides were analyzed with the Fluoview FV 1000 confocal microscope and Fluoview Viewer software (Olympus, Rungis, France).

### Western blotting

Whole cell lysates and western blotting were prepared as previously described [12]. Using the Cell Fractionation Kit (#9038, Cell Signaling Tech.), we separated cells into cytoplasmic (c), membrane/organelle (m), and nuclear/cytoskeletal (n) fractions and prepared the corresponding protein extracts according to the manufacturer’s instructions. We used primary Abs against β-actin (sc-47,778, final dilution 1/1000), cyclin D1 (sc-718, final dilution 1/400), cyclin D2 (sc-593, final dilution 1/200), p53 (sc-126, final dilution 1/400), p16^INK4A^ (sc-468, final dilution 1/100), and p21^CIP1^ (sc-397, final dilution 1/400) from Santa Cruz Biotech. (Santa Cruz, CA). We purchased Abs against MCL1 (#4572, final dilution 1/500), p-T256-cyclin D1 (#3300, final dilution 1/1000), CHK2 (#6334, final dilution 1/200), p-T68-CHK2 (#2197, final dilution 1/1000), p-S15-p53 (#9286, final dilution 1/1000), βTrCP1 ((#4394, final dilution 1/1000) from Cell Signaling Tech. (Danvers, MA). An Ab against BCL2 (clone 124, M0887, final dilution 1/200) was purchased from Dako (Glostrup, Denmark); an Ab against γH2AX (GTX61796, final dilution 1/1000) from Genetex (Irvine, CA); an Ab against GAPDH (clone 6C5, final dilution 1/2000) from Ambion (Thermo Fischer Scientific, Waltham, MA). We used ImmunoPure goat anti-rabbit or rabbit anti-mouse IgG peroxidase-conjugated as secondary Abs (Pierce, Thermo Fisher Scientific). For densitometric analyses, images were captured with a ChemiDoc™ XRS+ molecular imager and analyzed using Image Lab™ software (Bio-Rad). The background of each image was subtracted from the bands of interest, then the densities of each protein of interest were normalized against the density of control housekeeping proteins.

### Proteasome function assays

Our procedure was adapted from Vlashi et al. [14]. Cells were washed with PBS, pelleted and lysed in a homogenization buffer (25 mM Tris pH 7.5, 100 mM NaCl, 5 mM ATP, 0.2% (vol/vol) NP-40 and 20% glycerol). Cell debris were removed by centrifugation at 4 °C. Protein concentration in the resulting crude cellular extracts was determined. To measure 26S proteasome activity, 100 μg of protein of each sample were diluted with buffer I (50 mM Tris pH 7.4, 2 mM dithiothreitol, 5 mM MgCl_2_, 2 mM ATP) to a final volume of 1 ml and assayed in triplicate. The fluorogenic proteasome substrates Suc-LLVY-AMC (chymotryptic substrate; Enzo Life Sciences, Villeurbanne, France), Z-ARR-AMC (tryptic substrate; Calbiochem, Molsheim, France), and Z-LLE-AMC (caspase-like substrate; Enzo Life Sci.) were dissolved in DMSO and added to a final concentration of 80 μM. Proteolytic activities were continuously monitored for 120 min by measuring the release of the fluorescent group, 7-amido-4-methylcoumarin (AMC), with the use of a fluorescence plate reader (VICTOR X4 multilabel plate reader, Perkin Elmer) at 37 °C, at excitation and emission wavelengths of 380 and 460 nm, respectively. For analyzing the effects of proteasome/protease inhibitors on proteasome activities, cells were treated for 4 h with MG-132 (500 nM), bortezomib (5 nM) or leupeptin (20 μM) before the purification of whole cell extracts.

### RNA extraction and real-time polymerase chain reaction

Total RNAs were extracted using RNAeasy® Mini kit (Qiagen, Venlo, The Netherlands) according to the manufacturer’s instructions and quantified using a Smartspec™ 3000 spectrometer (Bio-Rad, Hercules, CA) from cultured MCL cells. cDNAs were synthesized using 2 μg of RNA and M-MuLV-reverse transcriptase as recommended by the supplier (Invitrogen, Thermo Fisher Scientific). SYBR Green real-time polymerase chain reaction (RT-PCR, Applied Biosystems, Thermo Fisher Scientific) was performed on cDNAs with primers for *BTRC* and *FBXW11* previously described [15], using a StepOnePlus real-time PCR System (Applied Biosystems). Data were analyzed with the Step One software V2.2.2 (Applied Biosystems). Gene expression was determined by real-time RT-PCR and quantified using *GAPDH* expression as internal standard. Relative gene expression was evaluated by the 2^-ΔΔCt^ method.

### Statistical analysis

The Student’s *t*-test was used to determine the significance of differences between two experimental groups. Data were analyzed by two-sided tests, with *p* < 0.05 (*) considered to be significant.

## Results

### MCL cell lines demonstrate differences in sensitivity to genotoxic agents

**Fig. 1:**
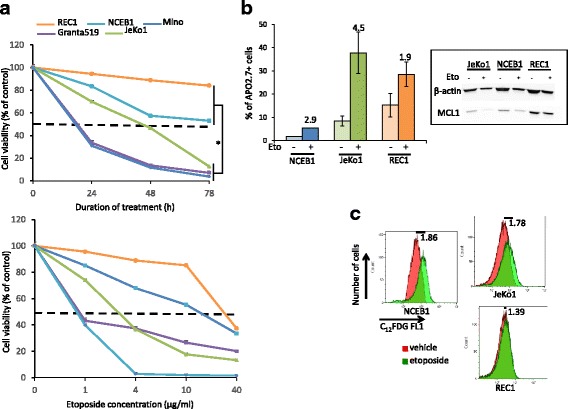
MCL cell lines are either sensitive or resistant to etoposide treatment. **a**. Exponentially growing Granta519, JeKo1, MINO, NCEB1 and REC1 cells were seeded at 5 × 10^4^ cells/well in 96-well plates and treated with vehicle, as a control, or 1 μg/ml etoposide for 24–72 h (upper panel) or with various concentrations of etoposide (1–40 μg/ml) for 24 h (lower panel). The cell proliferation was analyzed using an MTS assay and calculated as the ratio of absorbance of etoposide-treated cells vs. vehicle-treated cells for each time point and concentration. The absorbance values at 492 nm were corrected by subtracting the average absorbance from the control wells containing “no cells”. The experiment was performed twice, each culture condition was done in triplicate. The comparison of sensitive (Granta519, MINO and JeKo1) and resistant (REC1, NCEB1) using the *t*-test was significant. *, *p* < 0.05. **b**. Left part, MCL cells were treated with vehicle or 4 μg/ml etoposide for 24 h, stained with an anti-APO2.7 PE-conjugated Ab and analyzed by flow cytometry. The experiment was performed twice (for NCEB1) or three times; at least 10,000 events were gated for each culture condition. Means ± SD of % of APO2.7+ cells are indicated in the histogram as well as the fold induction (FI) calculated as the ratio of % APO2.7+ in etoposide- vs. vehicle-treated cultures. Right part, whole cell proteins were extracted, 40 μg of proteins were loaded on gels, separated by SDS-PAGE, blotted onto nitrocellulose membranes, incubated with Abs anti-MCL1, anti-BCL2 or anti-β-actin (as a loading control). **c**. MCL cells were treated with 4 μg/ml etoposide for 24 h or vehicle, as a control, then stained with C_12_FDG and analyzed by flow cytometry. At least 10,000 events were gated for each culture condition. The ratio mean of fluorescence intensity of vehicle vs. etoposide-treated cells is indicated on the figure

Etoposide, an inhibitor of topoisomerase 2 (Top 2), induces DSBs and triggers apoptosis. The response of five MCL cell lines (Granta519, Mino, REC1, NCEB1 and JeKo1) to etoposide was determined by a MTS cell proliferation assay. Cell lines were either exposed to vehicle or 1 μg/ml etoposide for 24–72 h or increasing concentrations of etoposide (1–40 μg/ml) for 24 h. Etoposide-treatment led to a dose- and time-dependent inhibition of proliferation (Fig. 1A). However, MCL cells displayed various sensitivities and in particular, REC1 and NCEB1 were more resistant than JEKO1, Mino and Granta519 cells (*p*-value = 0.0134 with the *t*-test). Using the PRISM 6 software, we analyzed the MTS data and calculated the IC_50_ and the area under the curve (AUC) for each cell line, for each treatment condition (Additional file 1: Figure S1). The gradient of sensitivity/resistance among the cell lines was confirmed (Additional file 2: Tables S1 and S2). We next selected three cell lines: REC1 (etoposide-resistant), NCEB1 (intermediate response) and JeKo1 (etoposide-sensitive) for studying the molecular basis of these responses.

### REC1 cells do not enter apoptosis or senescence after genotoxic stress

We analyzed the effects of etoposide on the induction of cell apoptosis. MCL cells were treated with 4 μg/ml etoposide for 24 h and apoptosis assayed after APO2.7 staining and flow cytometry analysis. Although JeKo1 cells underwent apoptosis (fold induction (FI) = 4.5), NCEB1 and REC1 cells did not show a similar magnitude (FI = 2.9 and 1.9, respectively) (Fig. 1B). In agreement with this observation, the level of MCL1 was gradually downregulated in MCL cells. The extent of downregulation was estimated by densitometry imaging and correlated with the amplitude of apoptototic response (Additional file 2: Table S3). MCL1 protein degradation is mandatory for MCL cell death [16]. In turn, the resistance of REC1 to genotoxic stress could be due to a defect of MCL1 degradation.

Following DNA damage, DDR is coordinated by ATM and ATR that phosphorylate multiple downstream targets and lead finally to cell cycle arrest, DNA repair or apoptosis [17]. DNA damage could also trigger an irreversible arrest of cell proliferation known as senescence [18]. We used C_12_FDG, a membrane-permeable molecule that stains senescent cells [13] for analyzing cell response after etoposide treatment. The number of C_12_FDG-stained NCEB1 and JeKo1 but not REC1 cells increased after treatment (Fig. 1C). To induce the long-term cell cycle arrest that accompanies senescence, two cell cycle inhibitors need to be up-regulated, p21^CIP1^ and p16^INK4A^. The level of both proteins was enhanced in etoposide-treated NCEB1 but not in REC1 cells (data not shown). These results showed that REC1 cells escaped both senescence and apoptosis following genotoxic stress.

### Etoposide activates DDR in MCL cells

**Fig. 2:**
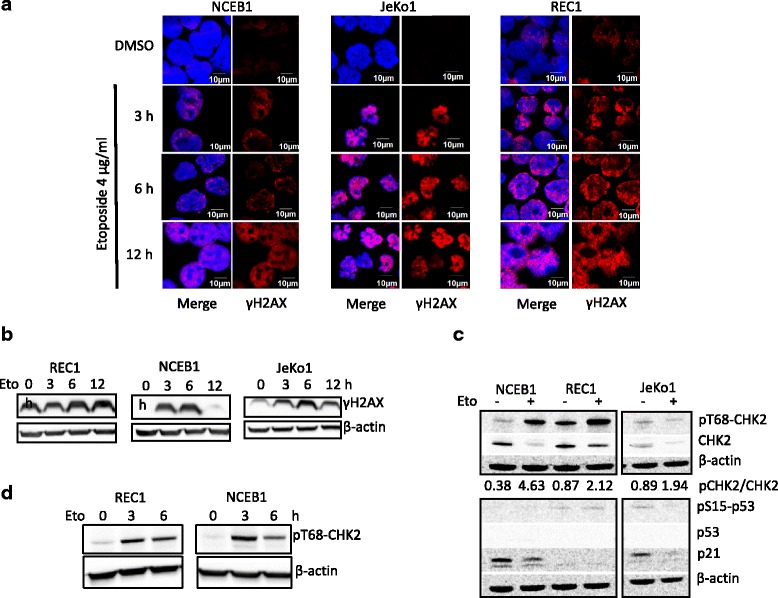
Etoposide activates DDR in MCL cells. **a**. NCEB1, JeKo1 and REC1 cells were treated with etoposide (4 μg/ml) or DMSO, as a control, and harvested 3, 6 or 12 h later. Cells were then processed for ICF with anti-γH2AX Ab, counterstained with DAPI and analyzed by confocal microscopy. **b**. MCL cells were treated with etoposide (4 μg/ml) or vehicle (0). Whole cell proteins were purified, 40 μg of proteins were loaded on gels, separated by SDS-PAGE, transferred onto nitrocellulose membranes and incubated with anti-γH2AX Ab. An anti-β-actin Ab served as a control for gel loading and transfer. **c**. MCL cells were treated with 4 μg/ml etoposide for 24 h and harvested. Western blots were performed as in B. Membranes were incubated with Abs anti-pT68-CHK2, −CHK2 and -β-actin (upper part). The density of specific bands was measured by densitometry and the ratio pCHK2/CKH2 indicated under the blots. Lower part, membranes were incubated with anti-pS15-p53, −p53, −p21 and -β-actin Abs as already described. **d**. Cultured REC1 and NCEB1 cells were treated as in b and western blots were done as described using Abs anti-pT68-CHK2 and -β-actin

**Fig. 3:**
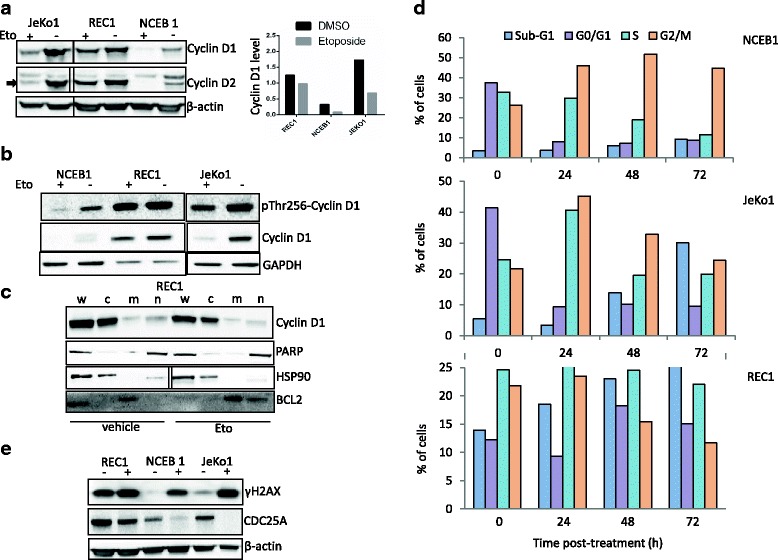
Cyclin D1 accumulates after genotoxic stress in REC1 cells. **a**. JeKo1, REC1, NCEB1 cells were treated with 4 μg/ml etoposide for 24 h or vehicle, as a control, and harvested. Western blots were performed as described in the legend of Fig. 2 using Abs anti-cyclin D1, −cyclin D2 and -β-actin Abs. The anti-cyclin D2 Ab detects also cyclin D1, the specific cyclin D2 band is arrowed on the figure. The level of cyclin D1 in vehicle and etoposide conditions was estimated by densitometry and reported on the graph. **b**. Cells were cultured and treated as before and analyzed by western blot with the indicated Abs. Anti-GAPDH Ab served as a control for gel loading and transfer. **c**. REC1 cells were treated with 4 μg/ml etoposide for 24 h or vehicle, as a control, and harvested. We purified proteins either from whole cell extracts (w), or from cytoplasm (c), membranes (m) and nucleus (n) compartments. After SDS-PAGE separation and membrane transfer, blots were incubated with an anti-cyclin D1 Ab. The purity of each fraction was verified with Abs specific for cytosolic protein (HSP90), membrane protein (BCL2) and nuclear protein (PARP). **d**. NCEB1, JeKo1 and REC1 cells were treated with vehicle (0) or 4 μg/ml etoposide for 24–72 h. The cells were stained with PI and analyzed by FACS (Gallios, Beckman Coulter). Data were processed with the Kaluza software (Beckman Coulter). The percentage of cells within each phase of the cell cycle (sub-G1, G0/G1, S and G2/M) is indicated on the histogram. At least, 10^4^ events were gated for each cell for each culture condition. The experiment has been carried out twice. **e**. MCL cells were treated with vehicle (−) or 4 μg/ml etoposide for 24 h (+) and harvested. Western blots were done as before with the indicated Abs

The resistance of REC1 cells to genotoxic stress could be due to alterations of DDR pathway. DSBs activate ATM/ATR kinases which leads to the phosphorylation of histone H2AX on Ser139 residues and generates γH2AX [19]. ATM/ATR apical kinases activate also DNA damage checkpoints to arrest cell cycle progression for repairing DNA [20]. We first evaluated the extent of DSBs generated following genotoxic stress. Immunocytofluorescence (ICF) analyses were performed after a 24-h treatment with 4 μg/ml etoposide. DSBs were absent in vehicle-treated NCEB1 and JeKo1 cells, and induced by etoposide-treatment (Fig. 2a). In contrast, vehicle-treated as well as untreated REC1 cells presented γH2AX foci representative of ongoing DNA damage (Fig. 2a, b and Fig. 3e). We next examined the kinetics of γH2AX generation in cells treated with 4 μg/ml etoposide by western blotting. γH2AX foci present in vehicle-treated REC1 cells progressed not much after etoposide treatment (Fig. 2b). In NCEB1 and JeKo1 cells, the level of γH2AX protein increased until 6 h then decreased indicating that DSBs were indeed repaired (Fig. 2b). This result showed that HR and/or NHEJ mechanisms of DNA repair were fully functional in NCEB1 and JeKo1 cells. However, according to Williamson et al. [21], NCEB1 and JeKo1 cells are *TP53*-mutated (Additional file 2: Table S4 and Fig. 2c) and in turn, unable to repair all DNA breaks driving cells to death. Interestingly, apoptosis induced in these responsive cells is p53-independent. DSBs were also able to activate CHK2 as observed by the phosphorylated state of the protein in REC1 cells (Fig. 2c). In that case, as shown both by the presence of γH2AX foci before any genotoxic treatment and after (Fig. 2a, b and Fig. 3e), and the phosphorylation of CHK2 (Fig. 2d), no DNA repair occurred. We concluded that DDR was activated in MCL cell lines including REC1 cells. However, DNA repair mechanisms were inefficient or incomplete in REC1 cells.

### Apoptotic response after genotoxic stress necessitates the downregulation of cyclin D1 and a block at the G2/M checkpoint

Accumulation of cyclin D1 is associated with genomic instability trough relicensing of DNA replication origins, DNA re-replication, DSB checkpoint activation [22, 23]. We next analyzed cyclin D1 expression in MCL cell lines treated with 4 μg/ml etoposide for 24 h. We observed a dramatic decrease of cyclin D1 level in JeKo1 and NCEB1 not in REC1 cells (Fig. 3a, upper part). This result was confirmed by densitometry (Fig. 3a, lower part). The downregulation of cyclin D1 was not accompanied by a compensatory upregulation of cyclin D2 (Fig. 3a). To be degraded by the proteasome/ubiquitin pathway, cyclin D1 needs to be phosphorylated (p-) on threonine 286 residue [24]. We then analyzed the phosphorylation status of cyclin D1 in etoposide- (4 μg/ml for 24 h) and vehicle-treated cells. As expected, phosphorylated forms of cyclin D1 were present in MCL cells including REC1 cells; p-cyclin D1 was downregulated as cyclin D1 in NCEB1 and JeKo1 cells following genotoxic insult not in REC1 cells (Fig. 3b). The absence of cyclin D1 degradation does not rely on a default of phosphorylation nor nuclear export. In REC1 cells, cyclin D1 resided in the cytosolic portion, mitochondrial membranes and nucleus as we reported previously for mature B cells [25]. Cyclin D1 shuttled into the cytosol after generation of DSBs for degradation by the ubiquitin/proteasome system (UPS). After etoposide treatment, we observed no accumulation of cyclin D1 into the nucleus, indicating that nuclear export occurred in REC1 cells ruling out abnormalities of the *XPO1* gene and/or the exportin 1 (Fig. 3c and Additional file 3: Figure S2A). We can speculate that, such as MCL1, cyclin D1 degradation could be mandatory for MCL cells to enter apoptosis and/or senescence. To confirm this point, we used doxorubicin in experimental conditions in which DSBs were generated without triggering apoptosis. Doxorubicin-treated MCL cells (25 nM for 24 h) exhibited activation of DDR (Additional file 3: Figure S2B). Moreover, MCL1, CDC25A, and cyclin D1 proteins were not degraded and, in turn, cells did not undergo apoptosis (Additional file 3: Figure S2B and not shown).

Cyclin D1 governs the cell cycle through the G1-S checkpoint. We analyzed the effects of etoposide treatment (4 μg/ml for 24–72 h) on cell cycle distribution after PI staining and flow cytometry analysis. As soon as 24 h post-treatment, NCEB1 cells were blocked at the G2 phase of the cell cycle and JeKo1 cells at both S and G2 phases (Fig. 3d). This block in the cycle progression was followed by the appearance at 48 and 72 h post-treatment of apoptotic cells (*i.e* cells with a sub-G1 DNA content) according to the magnitude of apoptosis. This cell cycle arrest did not occur in REC1 cells (Fig. 3d). Following genotoxic stress, REC1 cells escaped the G2/M block which precedes apoptosis.

CDC25A phosphatase is an essential regulator of G2/M transition and its degradation in response to DNA damage is critical for cell cycle arrest [26]. After activation of ATM/ATR and phosphorylation of CHK1/2, CDC25A became hyperphosphorylated and thus, degraded through its ubiquitylation. We next analyzed the level of CDC25A in etoposide- (4 μg/ml for 24 h) and vehicle-treated cells. CDC25A was not degraded in response to DNA damage in REC1 cells although the protein disappeared almost totally in NCEB1 and JeKo1 cells (Fig. 3e). In agreement with these results the level of CDC25A was maintained both in JeKo1 and REC1 cells following doxorubicin treatment (Additional file 3: Figure S2B).

### Cell response after DNA damage depends on the proteasome-ubiquitin pathway

**Fig. 4:**
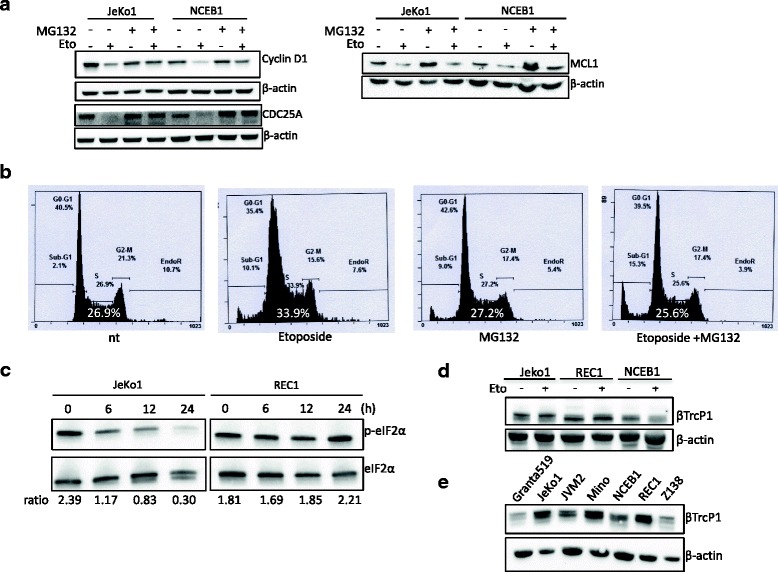
Cell response depends on the proteasome-ubiquitin pathway. **a**. JeKo1 and NCEB1 cells were treated with 1 μM etoposide, or 5 μM MG132, or both for 24 h, or vehicle, as a control, and harvested. Western blots were performed with the indicated Abs as described in the legend of Fig. 2. **b**. Cells were treated as in a, stained with PI and analyzed by flow cytometry. Cytometry profiles from a representative experiment are shown, the percentage of cells in the S phase is indicated on the graph. **c**. JeKo1 and REC1 cells were treated with 4 μg/ml etoposide (or vehicle, 0) for the indicated periods (6–24 h). Whole cell proteins were purified and analyzed by western blotting with the indicated Abs. The ratio p-eIF2α/ eIF2α estimated by densitometry, is indicated under the blots. **d**. Cells were treated with vehicle (−) or etoposide 4 μg/ml (+) for 24 h, then harvested. Whole cell proteins were purified, separated by SDS-PAGE, transferred onto nitrocellulose membranes and immunoblotted with an antibody against βTrcP1 protein. **e**. Proteins were purified from the indicated MCL cell lines and analyzed by western blot as described in d

Collectively, our data suggested that REC1 cells were resistant to etoposide because three main actors of apoptosis, senescence and cell proliferation: cyclin D1, MCL1 and CDC25A failed to be degraded by the proteasome machinery. We verified that the inhibition of proteasome by the MG132 inhibitor allowed cyclin D1, CDC25A, and MCL1 to resist degradation in JeKo1 and NCEB1 cells (Fig. 4a). Furthermore, in JeKo1 cells, a MG132-treatment relieved cell cycle block at the S/G2 transition (Fig. 4b). Cyclin D1, MCL1 and CDC25A are all proteins with a short half-life that are targeted by SCF complexes. Looking for an E3 ubiquitin ligase that could be involved in the ubiquitylation of cyclin D1, MCL1 and CDC25A, we found βTrCP a good candidate. Indeed, if essentially four F-box proteins were shown to contribute to cyclin D1 degradation (FBXO4, FBXW8, SKP2 and FBXO31), βTrCP could allow for cyclin D1 ubiquitylation in some conditions [27] and through an unconventional recognition site, ^279^EEVDLACpT^286^ [28]. Moreover, βTrCP targets MCL1 for ubiquitylation and destruction [29, 30], and controls the degradation pathway of CDC25A following DNA damage [31]. CReP (constitutive reverter of eIF2α phosphorylation) is a protein phosphatase 1 (PP1) that targets the translation initiation factor eIF2α to promote the removal of stress-induced inhibitory phosphorylation and increase cap-dependent translation. CReP is targeted by βTrCP for degradation upon DNA damage [32]. In good agreement with our previous results, following etoposide treatment (4 μg/ml for 24 h), the phosphorylated form of eIF2α was degraded in JeKo1 cells over time but not in REC1 cells (Fig. 4c).

### The response of MCL cells to etoposide is not driven by the βTrCP E3 ligase

Humans have two βTrCP paralogs: βTrCP1 (also known as FBXW1 or BTRC) and βTrCP2 (also known as FBXW11) with indistinguishable biochemical properties. Indeed, they share 86% identical amino acids in the F-box responsible for substrate specificity [33]. The two proteins are encoded by distinct genes localized on 10q24.32 for *BTRC* and 5q35.1 for *FBXW11* [15, 34]. The expression of BTRC and FBXW11 mRNAs was studied by a semi-quantitative RT-PCR in our panel of MCL cell lines (Granta519, JeKo1, JVM2, Mino, NCEB1, REC1 and Z138 cells; Additional file 2: Table S5). The *FBXW11* gene was expressed at a basal low level; by contrast, *BTRC* gene was expressed in all tested cells although with various levels (data not shown). Some commercially available antibodies against βTrCP detect unrelated proteins in western blots [35]. However, we validated an antibody directed against the βTrCP1 protein using Granta519 and MINO cells transiently transfected with pcDNA3-Flag-βTrCP1 and −2 expression plasmids ([26], data not shown). Two forms of βTrCP1 were detected in JeKo1, NCEB1 and REC1 cells. Etoposide treatment had no effects on βTrCP1 level in MCL cells (Fig. 4d). Moreover, the analysis of βTrCP1 level in the panel of MCL cells revealed a heterogeneous expression with no correlation with the magnitude of response towards etoposide (Fig. 4e). These data suggested that βTrCP was not altered in REC1 cells and not involved in cells resistance.

### 26S proteasome activity is lowered in REC1 cells

**Fig. 5:**
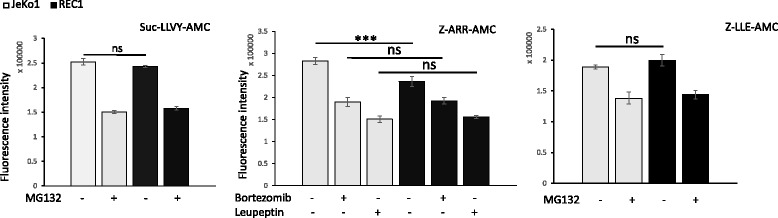
Proteasome activity is lowered in REC1 cells. JeKo1 and REC1 cells were treated with MG-132 (500 nM), bortezomib (5 nM), or leupeptin (20 μM) for 4 h or vehicle, and protein extracts were prepared. Chymotrypsin-, trypsin- and caspase-like activities were monitored using Suc-LLVY-AMC, Z-ARR-AMC, and Z-LLE-AMC fluorogenic substrates, respectively with a reader plate (Victor X4, Perkin Elmer). For all culture conditions and substrates, the protease activity reached a plateau at 60 min. Fluorescence intensities (in arbitrary unit) were taken at that time and presented in the histograms. The experiments were done three times with triplicate samples. ***, *p* < 0.001 with the *t*-test

The 26S proteasome is a macromolecular machinery composed of the 20S catalytic core and the 19S regulator [36]. The 20S core complex is shaped as a cylinder formed by a stack of four rings, each ring consisting in seven distinct subunits. The two outer rings are made of the α subunits (α1-α7); they mediate the interaction with the 19S regulatory core. The two inner rings are made of the β subunits (β1-β7); they possess at least three distinct proteolytic activities that can be monitored using specific fluorogenic substrates: Suc-LLVY-AMC, Z-ARR-AMC, and Z-LLE-AMC, respectively. β1, β2 and β5 subunits are referred to as having caspase-, trypsin-, and chymotrypsin-like activities based on their preference for cleaving peptides [36]. We further analyzed the 20S proteasome activity in cell lysates from either non-treated JeKo1 and REC1 cells or following a treatment with various proteasome inhibitors. In all cell extracts, chymotrypsin-, trypsin-, and caspase-like activities increased over time to reach a plateau at 60 min (data not shown). Therefore, we compared cells in various culture conditions at that time. As presented in Fig. 5 (left and right histograms), chymotrypsin and caspase-like activities were similar for JeKo1 and REC1 cells and inhibited by a MG-132 treatment. In agreement with our data, MG-132 binds with a high affinity the β5 site and a lower activity the β1 site of the proteasome [36]. In sharp contrast, the trypsin-like activity of the β2 site in REC1 cells was reduced compared to JeKo1 cells (*p* = 0.00083229 with the *t*-test). Whatever the initial activity, it was reduced to the same extent after bortezomib or leupeptin treatments (Fig. 5, middle panel). Bortezomib and leupeptin are both inhibitors of the trypsin-like activity [36]. These results showed that the trypsin-like activity of the 20S core particle is lowered in REC1 cells compared to JeKo1 cells.

## Discussion

Although therapeutic strategies for MCL have evolved these last years, the disease remains largely incurable. MCL patients develop *de novo* resistance or acquire resistance to frontline drugs [37]. There is a great need to overcome resistance in MCL patients and to improve their clinical outcome. This can be achieved through a better knowledge of the mechanisms of resistance. We studied the response of a panel of MCL cell lines to genotoxic stress and observed a heterogeneous response. REC1 cells are the most resistant and JeKo1 cells the most sensitive to etoposide, a Top 2 inhibitor that generates DSBs. We observed that three main actors of cell cycle arrest, senescence and apoptosis, namely cyclin D1, MCL1 and CDC25A that are enrolled after a genotoxic stress, fail to be degraded in response to etoposide. We finally provided evidence for a lowered 26S proteasome activity that could sustain the accumulation of these proteins and in turn, the resistance of MCL cells.

Defective proteolysis have been reported in MCL cells. Indeed, mutations of *CCND1* gene at the N-terminus increases cyclin D1 protein stability through the attenuation of threonine 286 phosphorylation and its nuclear retention [38]. The phosphorylation of cyclin D1 is mandatory for protein degradation by the UPS. We have shown that cyclin D1 is correctly phosphorylated in resistant REC1 cells after etoposide treatment and exported to the cytoplasmic compartment. Although E36K, Y44D, and C47S *CCND1* mutations have not been reported for REC1 cells, we can rule out such a mechanism of resistance. Importantly, in MCL cell lines, *CCND1* mutations promote resistance to ibrutinib, an inhibitor of the Bruton tyrosine kinase (BTK) involved in the B-cell receptor (BCR) signaling pathway. Recent studies have provided some clues about ibrutinib resistance including activation of the NF-κB, ERK1/2 and AKT, alteration of the BCR signaling pathways [39–41]. These studies suggest that multiple mechanisms contribute to ibrutinib resistance. Moreover, a same mechanism, i.e. the accumulation of nuclear cyclin D1, contributes to resistance to several drugs: ibrutinib [38], lenalidomide [12], and etoposide (the present study), drugs that target different pathways. According to these data, an increased stability of cyclin D1 is a major mechanism for MCL cells resistance.

REC1 cells are resistant to proteasome inhibitors: bortezomib (Additional file 3: Figure S2C, upper panel), MG132, and carfilzomib (data not shown)). However, REC1 cells enter apoptosis when treated with KNK-437, an inhibitor of heat-shock factor 1 (HSF1). HSF1 is the master transcription factor for heat shock proteins (HSPs) encoding genes. The inhibition of HSF1 downregulates simultaneously the transcription of *HSPB1* and *HSPA4* coding for HSP27 and HSP70, respectively. Acosta-Alvear and colleagues reported recently that proteostasis factors such as chaperones and HSPs controlled the response to proteasome inhibitors [42]. In particular, the knockdown of HSF1 sensitizes cells to carfilzomib in agreement with our observation. Moreover, the fact that REC1 cells respond to a HSF1 inhibitor suggests that the UPS although downregulated can be rearmed. Moreover, combining HSP inhibitors and proteasome inhibitors could be efficient therapies for MCL patients resistant to bortezomib/carfilzomib. Clinical trials have demonstrated such efficacy for bortezomib associated with several types of HSP90 [43–45] or HSF1 [46–48] inhibitors for multiple myeloma (MM) patients.

REC1 cells are sensitive to sn38, the metabolically active form of irinotecan, an inhibitor of Top 1, (Additional file 3: Figure S2C, lower panel). Importantly, the IC_50_ for sn38 is similar for REC1 and NCEB1 and smaller than JeKo1 cells. sn38 selectively targets Top 1-DNA cleavage complexes which form at the vicinity of replication and transcription complexes to unwind DNA. The stabilization of Top 1-DNA cleavage complexes leads to DNA damage at the time of DNA replication or transcription, and finally to DSBs [49]. DSBs either generated by sn38 or etoposide elicit the same DNA repair response. We showed in this study that DDR was activated in REC1 cells but that DNA repair mechanisms were deficient. This data is confirmed by the triggering of apoptosis after sn38-treatment in REC1 cells, in which the apoptotic machinery is fully functional. There is no cross-resistance in REC1 cells to Top 1 and Top 2 inhibitors. Moreover, the sensitivity/resistance pattern of MCL tumor cells towards drugs is multifactorial and largely dependent on the cell context.

Two studies reported recently a paradoxical resistance of multiple myeloma tumor cells to proteasome inhibitors by decreased levels of 19S proteasome regulatory sub-units [42, 50]. These are ATPase subunits as well as non-ATPase subunits and seem specific for a cell line. A downregulation of 19S sub-units could sustain the global decreased proteasome activity observed in REC1 cells and could be key determinant of resistance to proteasome inhibitors and other drugs in MCL tumor cells. However, the demonstration that the reduced trypsin-like activity bore by the 20S core complex and, in particular, the β2 subunit (or PSMB7), highlights another type of proteasome subunit abnormality. To our knowledge, this is the first report of a putative role of the β2 subunit in a resistance process. In contrast, the involvement of the β5 subunit in the resistance of various hemopathies is well described. For example, in a myelomonocytic THP1 cell line, selected for acquired resistance to bortezomib, the *PSMB5* gene coding for PSMB5, the β5 subunit, is mutated and the corresponding protein overexpressed [51]. In MM tumor cells resistant to bortezomib, no such mutations were found [52], rather a constitutive activation of the STAT3 signaling pathway and in turn, the upregulation of the β5 subunit [53]. However, the *PSMB7* gene coding for β2 subunit is overexpressed in a large variety of solid cancers and myeloid leukemias [54]. A survey of public available data bases (COSMIC, canSAR, Cancer Cell Line Ecyclopedia) indicated that *PSMB5* and *PSMB7* genes are not mutated, deleted nor amplified in REC1 cells. Further experiments should address these points.

Cancer stem cells (CSCs) or cancer-initiating cells (CICs) belong to a population of self-renewing cells that sustain the long-term clonal maintenance of the tumor [55]. Strong evidences support a link between stemness and resistance to drugs. CSCs/CICs have develop plethora of strategies to resist anticancer therapies including elevated activity for DNA damage detection and repair, increased ability for xenobiotic efflux, unbalance between the anti- vs. pro-apoptotic mechanisms, reduced production of free radicals etc. Interestingly, a low proteasome activity has been reported as a marker for breast cancer and head and neck CSCs/CICs [56, 57].

Several recurrent somatic mutations are described in tumor cells of MCL patients [58]. Among them, mutations of *ATM*, *CCND1*, *TP53*, *MLL2*, *TRAF2* and *NOTCH1* genes are frequently encountered and account for resistance to drugs. Most of them target the BCR and NF-κB signaling pathways and define actionable gene targets. However, deletion of *FBXO25* [9], mutation of *UBR5* [10] or, as suggested here, a decreased of global 26S proteasome activity modify the UPS and in turn, the sensitivity to drugs and the clinical response of MCL patients. Since, the resistance to proteasome inhibitor and/or other drugs is convoyed by defaults of UPS, the restoration of its activity seems determinant to bypass resistance and to achieve a full response towards treatments.

## Conclusions

MCL patients are either resistant or develop resistance after the treatments with frontline agents. Several mechanisms of resistance have been described in MCL tumor cells that escape apoptosis. We report here that a lowered 29S proteasome activity could be another mechanism of MCL cell resistance. Developing a strategy to counteract this mechanism of resistance may have significant therapeutic value.

## Additional files

Additional file 1: Figure S1. MCL cell lines were treated with vehicle or etoposide (10^−3^-10^2^ μg/ml) for 24–72 h. Cell viability was assayed using an MTS assay (CellTiter 96®AQ_ueous_ One Solution Cell Proliferation Assay, Promega). The absorbance (OD at 490 nM) of each clone treated with the drug is expressed relative to that of the corresponding clone treated with vehicle (defined as 100%). For each set of culture conditions, the mean ± SD of triplicate ratios is indicated on the curves. The experiment was performed twice. The results were analyzed with the PRISM® 6 software and reported in the Tables S1 and S2 in Additional file 1. (PDF 168 kb)

Additional file 2: Table S1. Calculation of IC_50_ for etoposide-treated MCL cell lines. **Table S2.** Calculation of AUC for etoposide-treated MCL cell lines. **Table S3.** MCL1 level is variously regulated following etoposide treatment. **Table S4.** Genetic characteristics of MCL cell lines. **Table S5.** Sequences of the primers used for RT-PCR. Additional references. (DOCX 120 kb)

Additional file 3: Figure S2. A. REC1 cells were treated with vehicle or etoposide 4 μg/ml for 2–24 h and harvested. Cells (10^5^ cells per spot) were cytospun on Superfrost glass slides, at 500 g for 3 min, then fixed in 4% paraformaldehyde (PFA) and permeabilized by incubation with 0.5% Triton-X100 (*v*/v) for 5 min. Slides were then stained with rabbit anti-cyclin D1 primary Ab (sc-718, Santa Cruz Biotech.) and AlexaFuor® 546 goat anti-rabbit IgG (Life Technologies) secondary Ab. DAPI (4′,6-diamidino-2-phenylindole dihydrochloride, Molecular Probes) served for nuclei counterstaining. Slides were mounted, and analyzed with a Fluoview FV 1000 confocal microscope and Fluoview Viewer software (Olympus). B. Cultured JeKo1 and REC1 cells were treated with vehicle (Ctrl) or doxorubicine (Dox 25 nM) for 24 h. Whole cell proteins were purified, separated by SDS-PAGE, and immunoblotted with the indicated antibodies. An anti-β-actin served as a control of charge and transfer. C. Cultured JeKo1 and REC1 cells were treated with vehicle (Ctrl) or bortezomib (10^−1^-10^4^ nM) or sn38 (10^−1^-10^3^ nM) for 24 h and then cell viability assessed by a MTS assay as described in the legend of the Fig. 1a. Dose-reponse curves were drawn with the PRISM® software (GraphPad, La Jolla, CA) and the IC_50_ were deduced from the data. (PPTX 5260 kb)

## Abbreviations

Ab: antibody
ampl: amplified
ATM: ataxia telangectasia mutant
ATR: ATM and Rad3-related
BCL2: B-cell lymphoma 2
BCR: B-cell receptor
BTK: Bruton tyrosine kinase
C_12_FDG: 5-dodecanoylaminofluorescein di-β-D-galactopyranoside
CDC25A: cell division cycle 25 homolog A
cDNA: complementary DNA
CHK1/2: checkpoint kinase 1/2
CIC: Cancer-initiating cell
CReP: constitutive reverter of eIF2α phosphorylation
CSC: cancer stem cell
DDR: DNA damage response
del: deleted
DSB: double-strand break
eIF2α: eukaryotic translation initiation factor 2α
hom: homozygous
HR: homologous recombination
HSP: heat shock protein
ICF: immunocytofluorescence
MCL: mantle cell lymphoma
MCL1: myeloid cell leukaemia sequence 1
MM: multiple myeloma
MTS: (3-(4,5-dimethylthiazol-2-yl)-5-(3-carboxymethoxyphenyl)-2-(4-sulfophenyl)-2H–tetrazolium)
mut: mutated
nd: not determined
NHEJ: non-homologous end-joining
p-: phosphorylated
PI: propidium iodide
PP1: protein phosphatase 1
qRT-PCR: quantitative reverse-transcriptase polymerase chain reaction
QVD-OPH: broad spectrum caspase inhibitor
SCF: SKP1/Cullin/F-box protein containing complex
SDS-PAGE: sodium dodecyl sulphate polyacrylamide gel electrophoresis
Top: topoisomerase
upd: uniparental disomy
UPS: ubiquitin proteasome system
wt: wild-type
βTrCP: β-transducin repeat containing protein
