# Supplementary figures and images for: A lowered 26S proteasome activity correlates with mantle lymphoma cell lines resistance to genotoxic stress
Source: BMC Cancer. 2017 Aug 10;17:538. doi: 10.1186/s12885-017-3530-z (PMC5553741; doi:10.1186/s12885-017-3530-z)

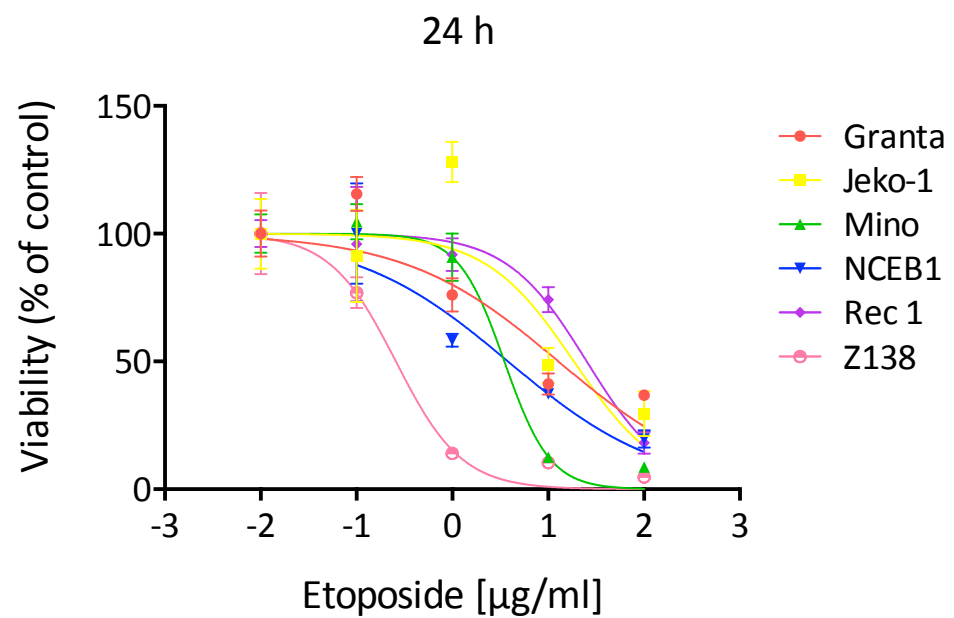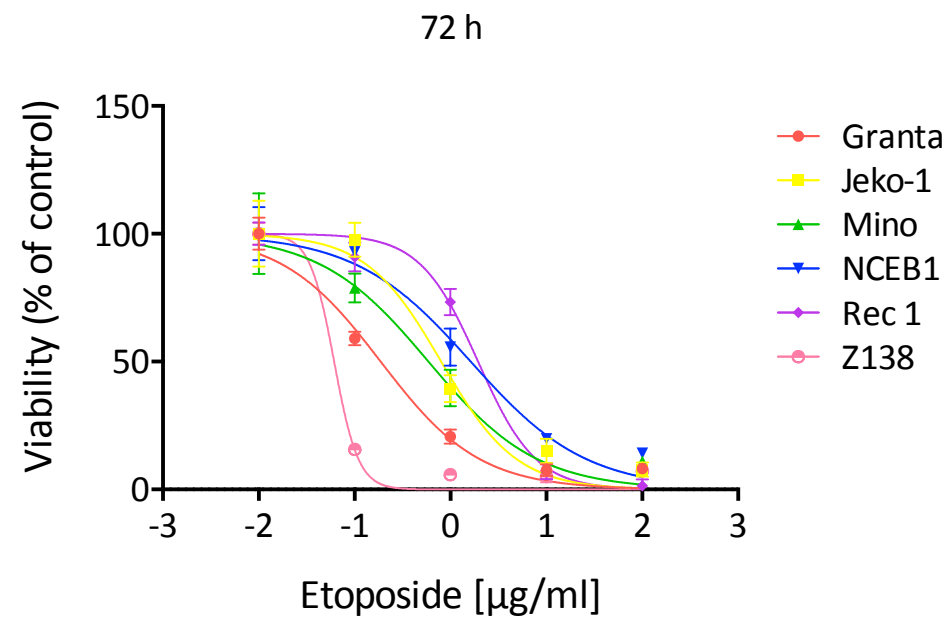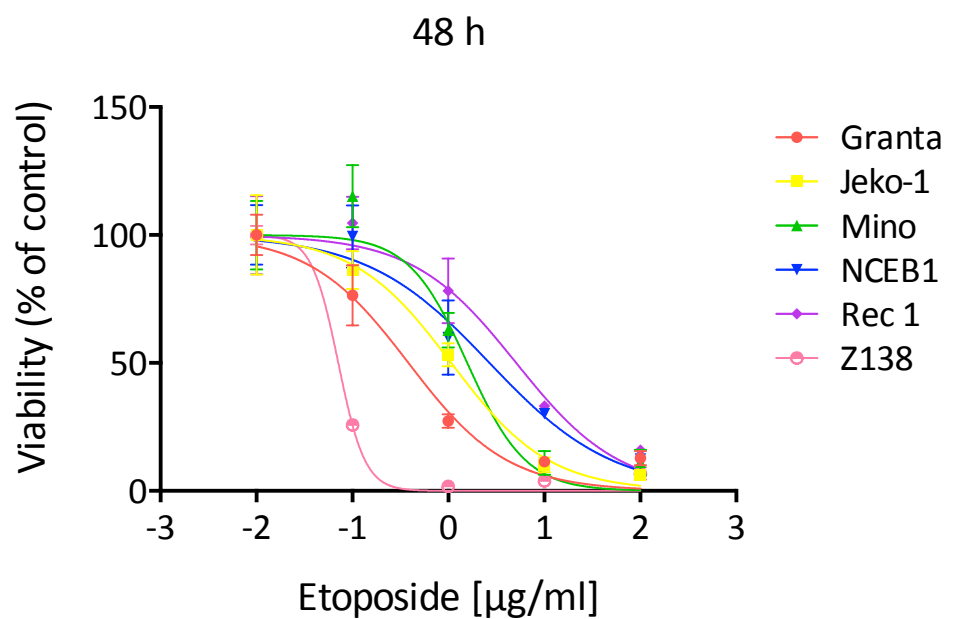

Figure S1

Supplement: Supplementary file 1 — MCL cell lines were treated with vehicle or etoposide (10−3-102 μg/ml) for 24–72 h. Cell viability was assayed using an MTS assay (CellTiter 96®AQueous One Solution Cell Proliferation Assay, Promega). The absorbance (OD at 490 nM) of each clone treated with the drug is expressed relative to that of the corresponding clone treated with vehicle (defined as 100%). For each set of culture conditions, the mean ± SD of triplicate ratios is indicated on the curves. The experiment was performed twice. The results were analyzed with the PRISM® 6 software and reported in the Tables S1 and S2 in Additional file 1. (PDF 168 kb) [file 12885_2017_3530_MOESM1_ESM.pdf]
