# Supplementary material for: A lowered 26S proteasome activity correlates with mantle lymphoma cell lines resistance to genotoxic stress
Source: BMC Cancer. 2017 Aug 10;17:538. doi: 10.1186/s12885-017-3530-z (PMC5553741; doi:10.1186/s12885-017-3530-z)
Supplement: Supplementary file 2 — Calculation of IC50 for etoposide-treated MCL cell lines. Table S2. Calculation of AUC for etoposide-treated MCL cell lines. Table S3. MCL1 level is variously regulated following etoposide treatment. Table S4. Genetic characteristics of MCL cell lines. Table S5. Sequences of the primers used for RT-PCR. Additional references. (DOCX 120 kb) [file 12885_2017_3530_MOESM2_ESM.docx]

**Additional file 1**

**Table S1.** Calculation of IC_50_ for etoposide-treated MCL cell lines

| Line | Granta | | JeKo1 | | Mino | | NCEB1 | | REC1 | | Z138 | |
| --- | --- | --- | --- | --- | --- | --- | --- | --- | --- | --- | --- | --- |
|  | IC_50_ | R^2^ | IC_50_ | R^2^ | IC_50_ | R^2^ | IC_50_ | R^2^ | IC_50_ | R^2^ | IC_50_ | R^2^ |
| 24 h | 12.80 | 0.8134 | 18.56 | 0.6932 | 3.50 | 0.9764 | 3.81 | 0.8629 | 15.38 | 0.9273 | 0.26 | 0.9601 |
| 48 h | 0.38 | 0.9473 | 1.07 | 0.9666 | 1.57 | 0.926 | 2.69 | 0.9268 | 3.46 | 0.9504 | 0.07 | 0.9882 |
| 72 h | 0.19 | 0.9695 | 0.80 | 0.9517 | 0.59 | 0.9522 | 1.63 | 0.9631 | 1.94 | 0.9856 | 0.06 | 0.9804 |

MCL cells were seeded in 96-well plates and treated with 10^-3^-10^2^ μg/ml etoposide for 24-72 h. Then, cell viability was assessed with an MTS assay (CellTiter 96®Aqueous One Solution Cell Proliferation, Promega). Data were analyzed with the PRISM® 6 software (GraphPad) and illustrated in Figure S1.

**Table S2.** Calculation of AUC for etoposide-treated MCL cell lines

| AUC | Granta | JeKo1 | Mino | NCEB1 | REC1 | Z138 |
| --- | --- | --- | --- | --- | --- | --- |
| 24 h | 301 | 332 | 262 | 255 | 321 | 154 |
| 48 h | 172 | 203 | 245 | 246 | 275 | 85 |
| 72 h | 141 | 206 | 182 | 222 | 225 | 80 |

MCL cells were analyzed as described in the legend of Table S1.

**Table S3**. MCL1 level is variously regulated following etoposide treatment

| Cell line | JeKo1 | | NCEB1 | | REC1 | |
| --- | --- | --- | --- | --- | --- | --- |
| Etoposide | - | + | - | + | - | + |
| MCL1 | 0.13 | 0.06 | 0.15 | 0.11 | 0.40 | 0.41 |

Exponentially growing MCL cells were treated with 4 μg/ml etoposide for 24 h (+) or with vehicle (-). Whole cell proteins were extracted, separated by SDS-PAGE, blotted onto nitrocellulose membranes, incubated with either anti-MCL1 or anti-β-actin (for gel loading control) antibodies. Blots were analyzed with a FluorSImager (Bio-Rad) and densitometric analyses with the Quantity One software (Bio-Rad). The ratio of MCL1 level *vs.* β-actin level was calculated and indicated in the table.

**Table S4**. Genetic characteristics of MCL cell lines

| MCL cell line | *TP53* | *ATM* | *CDKN2A* |
| --- | --- | --- | --- |
| Granta519 | del/wt | del/mut | hom del |
| JeKo1 | del/mut | ampl/nd | del/nd |
| Mino | upd/mut | wt/nd | upd/nd |
| NCEB1 | del/mut | del/nd | wt/nd |
| REC1 | mut/wt | wt/nd | hom del |
| Z138 | wt/wt | del/nd | hom del |

Data were compiled from refs. 1-3 and our data. Abbreviations: mut, mutated; del, deleted, wt, wild type; hom, homozygous; nd, not determined; ampl, amplified; upd, uniparental disomy.

**Table S5**. Sequences of the primers used for RT-PCR

|  |  | Primer sequences (5'-3') | |
| --- | --- | --- | --- |
| Gene symbol | Gene product | Forward | Reverse |
| *BTRC* | β-TrCP1 | atc gga ttc cac ggt cag ag | aat caa cgt gtt tag cat ttc acc t |
| *FBXW11* | β-TrCP2 | cca tca aag tct gga gca cga | cgc ttg tgc cca ttg aga gta |
| *GAPDH* | GAPDH | ctg act tca aca gcg aca cc | ccc tgt tgc tgt agc caa at |

**Additional references**

1. Williamson CT, Kubota E, Hamill JD, Klimowicz A, Ye R, Muzik H, Dean M, Tu L, Gilley D, Magliocco AM, Mckay BC, Bebb DG, Lees-Miller SP. Enhanced cytotoxicity of PARP inhibition in mantle cell lymphoma harbouring mutations in both ATM and p53. EMBO Mol Med 2012;4:515–27.

2. Xargay-Torrent S, López-Guerra M, Montraveta A, Saborit-Villarroya I, Rosich L, Navarro A, Pérez-Galán P, Roué G, Campo E, Colomer D. Sorafenib inhibits cell migration and stroma-mediated bortezomib resistance by interfering B-cell receptor signaling and protein translation in mantle cell lymphoma. Clin Cancer Res 2013;19:586–97.

3. Montraveta A, Xargay-Torrent S, López-Guerra M, Rosich L, Pérez-Galán P, Salaverria I, Beà S, Kalko SG, de Frias M, Campàs C, Roué G, Colomer G. Synergistic anti-tumor activity of acadesine (AICAR) in combination with the anti-CD20 monoclonal antibody rituximab in *in vivo* and *in vitro* models of mantle cell lymphoma. Oncotarget 2014;5:726–39.
