# Supplementary material for: A lowered 26S proteasome activity correlates with mantle lymphoma cell lines resistance to genotoxic stress
Source: BMC Cancer. 2017 Aug 10;17:538. doi: 10.1186/s12885-017-3530-z (PMC5553741; doi:10.1186/s12885-017-3530-z)
Supplement: Supplementary file 3 — A. REC1 cells were treated with vehicle or etoposide 4 μg/ml for 2–24 h and harvested. Cells (105 cells per spot) were cytospun on Superfrost glass slides, at 500 g for 3 min, then fixed in 4% paraformaldehyde (PFA) and permeabilized by incubation with 0.5% Triton-X100 (v/v) for 5 min. Slides were then stained with rabbit anti-cyclin D1 primary Ab (sc-718, Santa Cruz Biotech.) and AlexaFuor® 546 goat anti-rabbit IgG (Life Technologies) secondary Ab. DAPI (4′,6-diamidino-2-phenylindole dihydrochloride, Molecular Probes) served for nuclei counterstaining. Slides were mounted, and analyzed with a Fluoview FV 1000 confocal microscope and Fluoview Viewer software (Olympus). B. Cultured JeKo1 and REC1 cells were treated with vehicle (Ctrl) or doxorubicine (Dox 25 nM) for 24 h. Whole cell proteins were purified, separated by SDS-PAGE, and immunoblotted with the indicated antibodies. An anti-β-actin served as a control of charge and transfer. C. Cultured JeKo1 and REC1 cells were treated with vehicle (Ctrl) or bortezomib (10−1-104 nM) or sn38 (10−1-103 nM) for 24 h and then cell viability assessed by a MTS assay as described in the legend of the Fig. 1a. Dose-reponse curves were drawn with the PRISM® software (GraphPad, La Jolla, CA) and the IC50 were deduced from the data. (PPTX 5260 kb) [file 12885_2017_3530_MOESM3_ESM.pptx]

## Slide 1
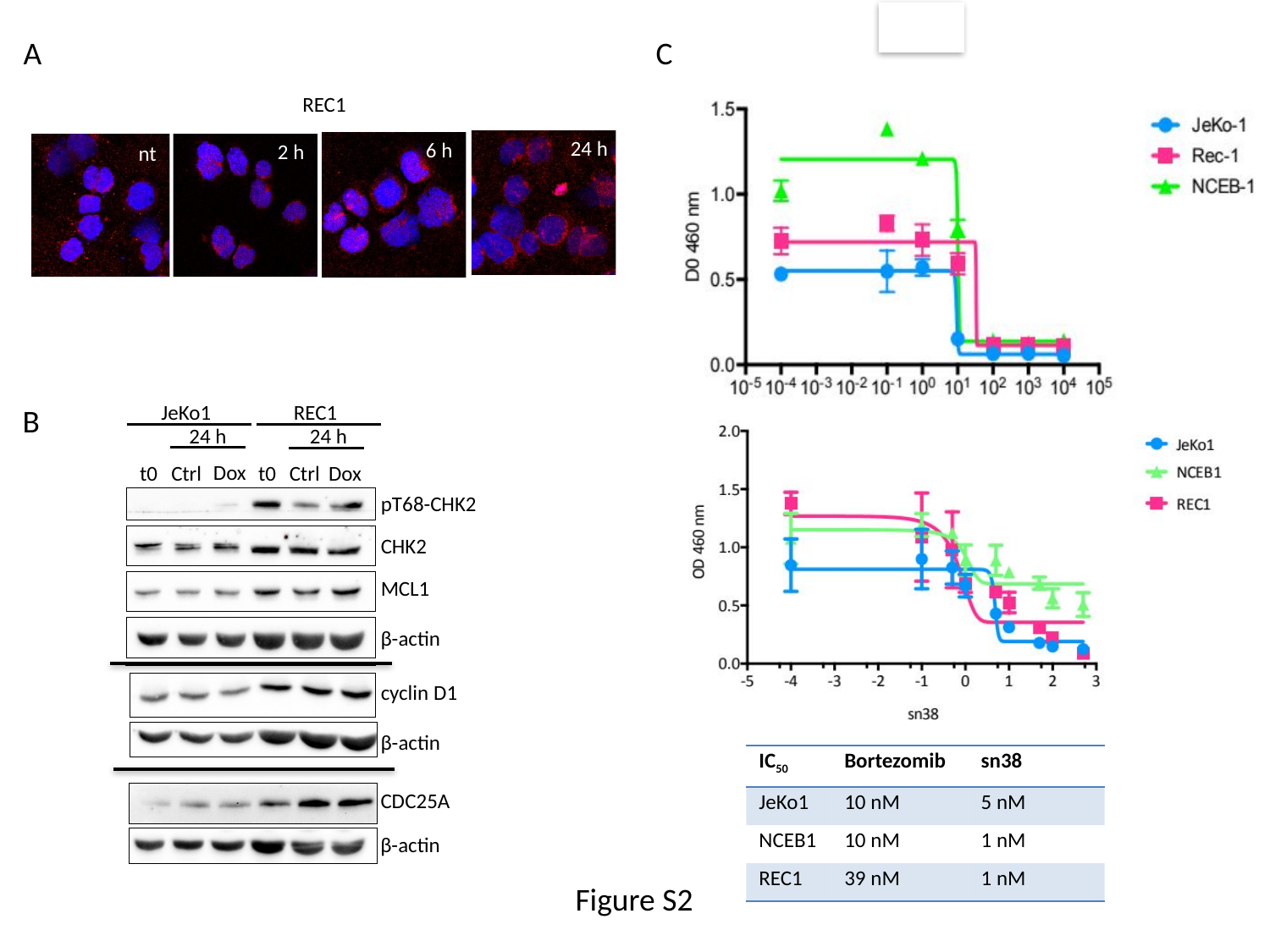

A
C
REC1
24 h
6 h
2 h
nt
JeKo1
REC1
24 h
24 h
Dox
t0
Dox
Ctrl
Ctrl
t0
pT68-CHK2
CHK2
MCL1
β-actin
cyclin D1
β-actin
B
| IC50 | Bortezomib | sn38 |
| --- | --- | --- |
| JeKo1 | 10 nM | 5 nM |
| NCEB1 | 10 nM | 1 nM |
| REC1 | 39 nM | 1 nM |
CDC25A
β-actin
Figure S2
